# Vascular endothelial growth factor A polymorphisms are associated with increased risk of coronary heart disease: a meta-analysis

**DOI:** 10.18632/oncotarget.15546

**Published:** 2017-02-20

**Authors:** Yafeng Wang, Qiuyu Huang, Jianchao Liu, Yanan Wang, Gongfeng Zheng, Ling Lin, Hui Yu, Weifeng Tang, Ziyang Huang

**Affiliations:** ^1^ Cardiovascular Department, The Second Clinical Medical College of Fujian Medical University, Quanzhou, Fujian, China; ^2^ Department of Cardiac Surgery, Union Hospital, Fujian Medical University, Fuzhou, Fujian, China; ^3^ Department of Cardiothoracic Surgery, Affiliated Peoples Hospital of Jiangsu University, Zhenjiang, Fujian, China; ^4^ Department of Function, Agribusiness Hospital of Xishuangbanna, Jinghong, Yunnan, China; ^5^ Rheumatism Department, The Second Clinical Medical College of Fujian Medical University, Quanzhou, Fujian, China; ^6^ Department of Abdominal Surgery, Fujian Medical University Cancer Hospital, Fuzhou, Fujian, China

**Keywords:** VEGFA, coronary heart disease, susceptibility, polymorphism, meta-analysis

## Abstract

Coronary heart disease (CHD) is a common complex disease resulting from the interaction of multiple environmental and genetic factors. To assess the potential relationship of vascular endothelial growth factor (*VEGFA*) rs699947 C>A, rs3025039 C>T and rs2010963 G>C polymorphisms with CHD risk, a comprehensive meta-analysis was conducted. A systematic search of EMBASE and PubMed online database for publications on *VEGFA* polymorphisms and risk of CHD was carried out. Crude Odds ratios (ORs) with their 95% confidence intervals (CIs) were calculated to determine the association. A total of ten publications including 22 trails involving 2097 cases and 2867 controls were included in our pooled analysis. Overall, results of the present meta-analysis demonstrated a significant association between *VEGFA* rs699947 C>A polymorphism and an increased risk of CHD. After stratifying by ethnicity and CHD type, the association was also obtained. A significant association between *VEGFA* rs3025039 C>T polymorphism and risk of CHD was also found. For *VEGFA* rs2010963 G>C polymorphism, the polymorphism was associated with MI risk. In conclusion, our findings suggest that *VEGFA* rs699947 C>A, rs3025039 C>T and rs2010963 G>C polymorphisms are risk factors for CHD. In the future, large sample size and well-designed epidemiologic studies are needed to confirm these conclusions.

## INTRODUCTION

Coronary heart disease (CHD) is one of the leading causes of mortality and morbidity worldwide [1, 2]. Besides environmental risk factors (e.g. smoking, drinking, and sedentary lifestyle *et al*.), genetic factors, such as single-nucleotide polymorphisms (SNPs), may play prominent roles in the development of CHD [3].

Vascular endothelial growth factor (VEGFA) is a glycoprotein molecule generated by the vascular endothelium, retinal pigment epithelium, pericytes, T cells and macrophages *et al* [4]. VEGFA, one of the most potent mitogens, acts as an important promoter of angiogenesis in both lymphogenesis and angiogenesis [5, 6]. It was reported that inflammation and neovascularization in atheromatous plaques might be mediated by VEGFA [7]. Previous study also found that increased plasma VEGFA levels in CHD patients may indicate the severity of coronary lesion, and it may be adopted as an indicator of the need for revascularization [8, 9]. These results suggested that VEGFA might be involved in the development of CHD.

The *VEGF* gene, also named as vascular permeability factor, is located on chromosome 6p21.3 and contains eight exons [10]. VEGF family consists of VEGFA, VEGFB, VEGFC, VEGFD, VEGFE, VEGFF and placental growth factor. The human *VEGFA* gene is very polymorphic (http://www.ncbi.nlm.nih.gov/SNP). And the variants of *VEGFA* gene may influence the expression between individuals [11]. Functional studies indicated that a number of variants in *VEGFA* gene were correlated with the level of mRNA and protein expression [12, 13]. Three single nucleotide polymorphisms (SNPs), *VEGFA* rs699947 (−2578C > A), rs3025039 (+936C > T) and rs2010963 G > C were extensively studied their associations with CHD; however, the results remained inconsistent. Recently, a systematic review and meta-analysis showed that *VEGFA* rs699947 polymorphism was not associated with CHD [14]. However, in this pooled analysis [14], only three case-control studies focusing on Caucasians were included, the power of this pooled-analyses might be insufficient. Of late, more epidemiologic studies with relatively large sample size focusing on the potential association of *VEGFA* rs699947 C > A,rs3025039 C > T and rs2010963 G > C polymorphisms with CHD risk were carried out. Considering the potential role of *VEGFA* rs699947 C > A, rs3025039 C > T and rs2010963 G > C polymorphism for CHD susceptibility, this coverage might increase the statistical power to assess the association of *VEGFA* rs699947 C > A, rs3025039 C > T and rs2010963 G > C polymorphisms with CHD risk.

## RESULTS

### Characteristics

**Figure 1:**
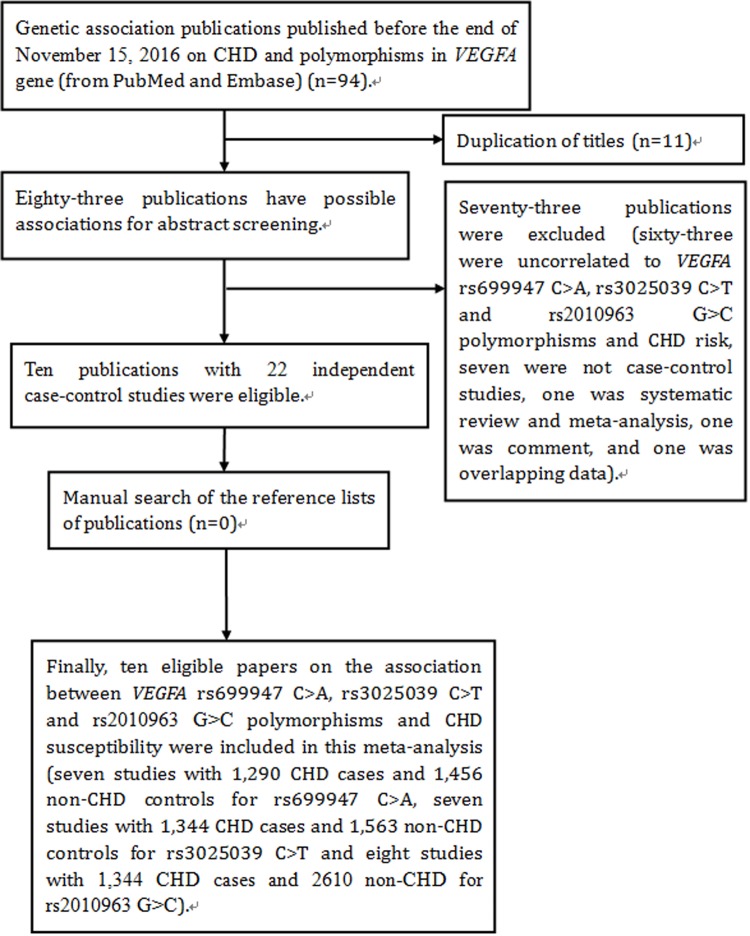
Flow diagram of studies selection

There were two independent groups in a paper conducted by Kangas-Kontio *et al*., we treated them separately [19]. According to the major inclusion and exclusion criteria, ten eligible publications with 22 independent case-control studies [19–28] were included to extract the data. The flow chart of the detailed publication selection is summarized in Figure 1. For *VEGFA* rs699947 C > A polymorphism, a total of 1,290 CHD cases and 1,456 non-CHD controls from seven independent case-control studies [19–24] were included in this meta-analysis. The year of publication ranged from 2008 to 2013. Two of these studies were conducted in Asians [20, 21] and five studies in Caucasians [19, 22–24]. Using a Goodness-of-fit chi-square calculator, the HWE test was performed; the genotype distributions of controls were all in HWE (*P* > 0.05). In total, for *VEGFA* rs3025039 C > T polymorphism, 1,344 CHD cases and 1,563 non-CHD controls from seven independent case-control studies were included [19–21, 24–26]. The year of publication ranged from 2008 to 2015. Three of these studies were conducted in Asians [20, 21, 26] and four studies in Caucasians [19, 24, 25]. The HWE test was conducted; the genotype distributions of controls were all in HWE (*P* > 0.05). And for *VEGFA* rs2010963 G > C polymorphism, 1,344 CHD cases and 2610 non-CHD controls from eight independent case-control studies were included [19–21, 25–28]. The year of publication ranged from 2006 to 2015. Three of these studies were conducted in Asians [20, 21, 26] and five studies in Caucasians [19, 25, 27, 28]. The HWE test was conducted; the genotype distributions of controls were all in HWE (*P* > 0.05). The characteristics of the included studies are shown in Table 1. The genotype distributions of the *VEGFA* rs699947 C > A, rs3025039 C > T and rs2010963 polymorphisms in CHD cases and controls are presented in Table 2, Table 3 and Table 4, respectively.

**Table 1:** Characteristics of the eligible studies in the meta-analysis

study	year	country	ethnicity	CHD type	No. of cases/controls	Genotype Method	polymorphisms
Han *et al*.	2015	China	Asians	coronary heart disease	144/150	MALDI-TOF MS	rs3025039 C>T and rs2010963 G>C
Moradzadegan et al.	2015	Iran	Caucasians	coronary heart disease	141/369	PCR-RFLP	rs2010963 G>C
Gu *et al*.	2013	China	Asians	coronary heart disease	435/480	MALDI-TOF MS	rs699947 C>A, rs3025039 C>T and rs2010963 G>C
Cui *et al*.	2013	China	Asians	coronary heart disease	242/253	MALDI-TOF MS	rs699947 C>A, rs3025039 C>T and rs2010963 G>C
Amoli *et al*.	2012	Iran	Caucasians	coronary heart disease	50/50	ARMS–PCR	rs699947 C>A
Guerzoni *et al*.	2009	Brazil	Caucasians	coronary heart disease	145/99	PCR-SSCP	rs699947 C>A
Douvaras *et al*.	2009	Greece	Caucasians	myocardial infarction	102/98	PCR-RFLP	rs3025039 C>T and rs2010963 G>C
Kangas-Kontio *et al*.	2009	Finland	Caucasians	myocardial infarction	215/218	TaqMan	rs699947 C>A, rs3025039 C>T and rs2010963 G>C
Kangas-Kontio *et al*.	2009	Finland	Caucasians	myocardial infarction	36/263	TaqMan	rs699947 C>A, rs3025039 C>T and rs2010963 G>C
Biselli *et al*.	2008	Brazil	Caucasians	coronary heart disease	175/108	PCR-SSCP	rs699947 C>A and rs3025039 C>T
Petrovic et al.	2006	Slovenia	Caucasians	myocardial infarction	143/228	PCR-RFLP	rs2010963 G>C

Abbreviations: MALDI-TOF MS, Matrix-Assisted Laser Desorption/Ionization Time of Flight Mass Spectrometry; ARMS-PCR, Amplification Refractory Mutation System-Polymerase Chain Reaction; PCR-SSCP, Polymerase Chain Reaction-Single-Strand Conformational Polymorphism; PCR-RFLP, Polymerase Chain Reaction -Restriction Fragment Length Polymorphism.

**Table 2:** Distribution of *VEGFA* rs699947 C>A polymorphism genotypes and alleles

study	year	Case genotype			Control genotype			Case allele		Control allele		HWE
		CC	CA	AA	CC	CA	AA	C	A	C	A	
Gu et al.	2013	219	178	30	267	174	31	616	238	708	236	YES
Cui et al.	2013	137	78	27	172	69	12	352	132	413	93	YES
Amoli et al.	2012	9	27	14	15	26	9	45	55	56	44	YES
Guerzoni et al.	2009	34	83	28	29	46	24	151	139	104	94	YES
Kangas-Kontio et al.	2009	36	104	75	40	101	70	176	254	181	241	YES
Kangas-Kontio et al.	2009	4	18	14	53	129	81	26	46	235	291	YES
Biselli et al.	2008	47	96	32	30	51	27	190	160	111	105	YES

Abbreviation: HWE, Hardy–Weinberg equilibrium

**Table 3:** Distribution of *VEGFA* rs3025039 C>T polymorphism genotypes and alleles

study	year	Case genotype			Control genotype			Case allele		Control allele		HWE
		CC	CT	TT	CC	CT	TT	C	T	C	T	
Han et al.	2015	84	55	5	115	31	4	223	65	261	39	YES
Gu et al.	2013	272	142	16	300	159	14	686	174	759	187	YES
Cui et al.	2013	133	95	14	159	86	8	361	123	404	102	YES
Douvaras et al.	2009	68	30	4	69	27	2	166	38	165	31	YES
Kangas-Kontio et al.	2009	160	50	5	155	56	7	370	60	366	70	YES
Kangas-Kontio et al.	2009	23	13	0	184	72	7	59	13	440	86	YES
Biselli et al.	2008	133	36	6	83	23	2	302	48	189	27	YES

Abbreviation: HWE, Hardy–Weinberg equilibrium

**Table 4:** Distribution of *VEGFA* rs2010963 G>C polymorphism genotypes and allelles

	year	Case genotype			Control genotype			Case allele		Control allele		HWE
		case GG	case GC	case CC	control GG	control GC	control CC	Case G	Case C	Control G	Control C	
Han et al.	2015	69	49	26	86	54	10	187	101	226	74	YES
Moradzadegan et al.	2015	43	65	33	85	197	87	151	131	367	371	YES
Gu et al.	2013	144	215	60	154	225	89	503	335	533	403	YES
Cui et al.	2013	75	102	65	104	114	35	252	232	322	184	YES
Douvaras et al.	2009	37	49	16	29	55	14	123	81	113	83	YES
Kangas-Kontio et al.	2009	132	72	10	143	67	8	336	92	353	83	YES
Kangas-Kontio et al.	2009	22	10	3	154	90	19	54	16	398	128	YES
Petrovic et al.	2006	42	76	25	103	104	21	160	126	310	146	YES

Abbreviation: HWE, Hardy–Weinberg equilibrium

### Quantitative synthesis

**Figure 2:**
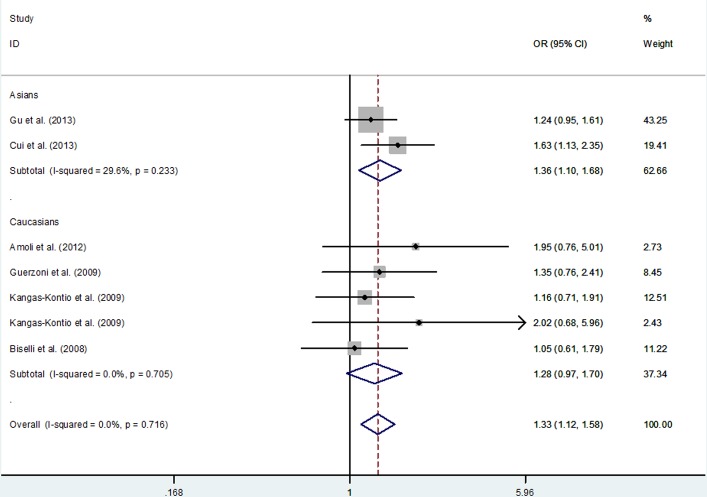
Meta-analysis for the association between *VEGFA* rs699947 C > A polymorphism and CHD risk (AA+CA *vs*. CC genetic model, fixed-effects model)

Overall, *VEGFA* rs699947 C > A polymorphism was a risk factor for CHD (A *vs*. C: OR = 1.19; 95% CI, 1.05 - 1.34; *P* = 0.005; AA *vs*. CC: OR = 1.33; 95% CI, 1.03-1.73; *P* = 0.032 and AA+CA *vs*. CC: OR = 1.33; 95% CI, 1.12-1.58; *P* = 0.001; Table 5 and Figure 2). In subgroup analyses by ethnicity, the similar association was found among Asians (AA+CA *vs*. CC: OR = 1.36; 95% CI, 1.10-1.68; *P* = 0.005; Table 5). In subgroup analyses by the type of CHD, *VEGFA* rs699947 C > A polymorphism was also associated with risk of non-MI (AA+CA *vs*. CC: OR = 1.34; 95% CI, 1.11-1.60; *P* = 0.002; Table 5).

**Table 5:** Meta-analysis of the *VEGFA* rs699947 C>A polymorphism and CHD

	No. of study	Allelic comparison			Homozygote comparison			Dominant comparison			Recessive comparison		
		OR(95%CI)	*P*	P(Q-test)	OR(95%CI)	*P*	P(Q-test)	OR(95%CI)	*P*	P(Q-test)	OR(95%CI)	*P*	P(Q-test)
Overall	7	**1.19(1.05-1.34)**	**0.005**	0.117	**1.33(1.03-1.73)**	**0.032**	0.131	**1.33(1.12-1.58)**	**0.001**	0.716	1.14(0.83-1.55)	0.422	0.085
Ethnicity													
Asians	2	1.37(0.96-1.95)	0.084	0.053	1.76(0.75-4.14)	0.192	0.055	**1.36(1.10-1.68)**	**0.005**	0.233	1.59(0.69-3.66)	0.275	0.056
Caucasians	5	1.09(0.92-1.28)	0.324	0.388	1.17(0.84-1.64)	0.361	0.311	1.28(0.97-1.70)	0.080	0.705	0.99(0.76-1.28)	0.947	0.273
Type of CHD													
MI	2	1.15(0.91-1.47)	0.242	0.349	1.36(0.83-2.24)	0.220	0.319	1.30(0.83-2.03)	0.245	0.362	1.15(0.81-1.64)	0.432	0.503
Non-MI	5	1.20(0.96-1.50)	0.108	0.055	1.36(0.84-2.21)	0.213	0.065	**1.34(1.11-1.60)**	**0.002**	0.582	1.13(0.71-1.82)	0.604	0.032

Abbreviations: MI: myocardial infarction;

CHD: coronary heart disease

**Figure 3:**
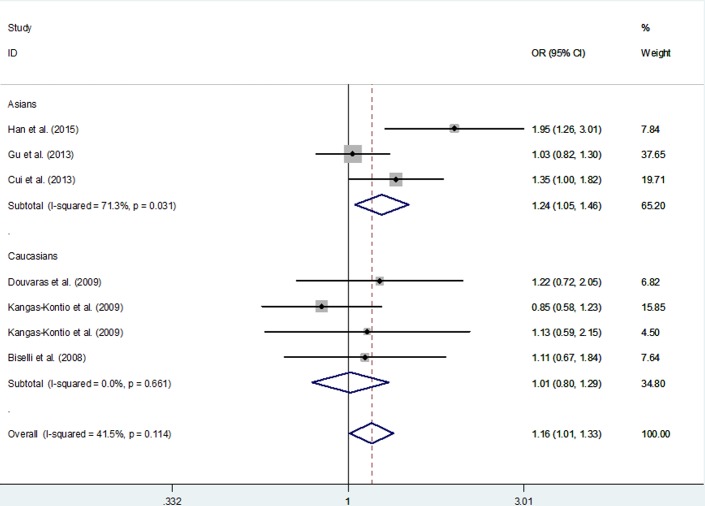
Meta-analysis for the association between VEGFA rs3025039 C > T polymorphism and CHD risk (T *vs*. C genetic model; fixed-effects model)

For *VEGFA* rs3025039 C > T polymorphism, this SNP was associated with increased risk of overall CHD in one genetic models (T *vs*. C: OR = 1.16; 95% CI, 1.01 - 1.33; *P* = 0.035; Table 6 and Figure 3). However, in a subgroup analysis by ethnicity and the type of CHD, the association was not identified (Table 6).

**Table 6:** Meta-analysis of the *VEGFA* rs3025039 C>T polymorphism and CHD

	No. of study	Allelic comparison			Homozygote comparison			Dominant comparison			Recessive comparison		
		OR(95%CI)	*P*	P(Q-test)	OR(95%CI)	*P*	P(Q-test)	OR(95%CI)	*P*	P(Q-test)	OR(95%CI)	*P*	P(Q-test)
Overall	7	**1.16(1.01-1.33)**	0.035	0.114	1.40(0.91-2.15)	0.125	0.800	1.21(0.95-1.55)	0.117	0.065	1.33(0.87-2.04)	0.189	0.862
Ethnicity													
Asians	3	1.34(0.96-1.87)	0.089	0.031	1.57(0.93-2.65)	0.089	0.687	1.42(0.91-2.22)	0.119	0.012	1.46(0.87-2.45)	0.149	0.786
Caucasians	4	1.01(0.80-1.29)	0.906	0.661	1.09(0.51-2.33)	0.825	0.626	1.02(0.77-1.33)	0.914	0.686	1.09(0.51-2.31)	0.832	0.635
Type of CHD													
MI	3	0.99(0.75-1.30)	0.926	0.490	0.91(0.38-2.20)	0.835	0.555	1.01(0.74-1.37)	0.974	0.480	0.91(0.38-2.17)	0.823	0.569
Non-MI	4	1.29(0.98-1.68)	0.065	0.068	1.60(0.98-2.63)	0.063	0.852	1.33(0.93-1.90)	0.113	0.027	1.50(0.92-2.45)	0.106	0.904

Abbreviations: MI: myocardial infarction;

CHD: coronary heart disease

**Figure 4:**
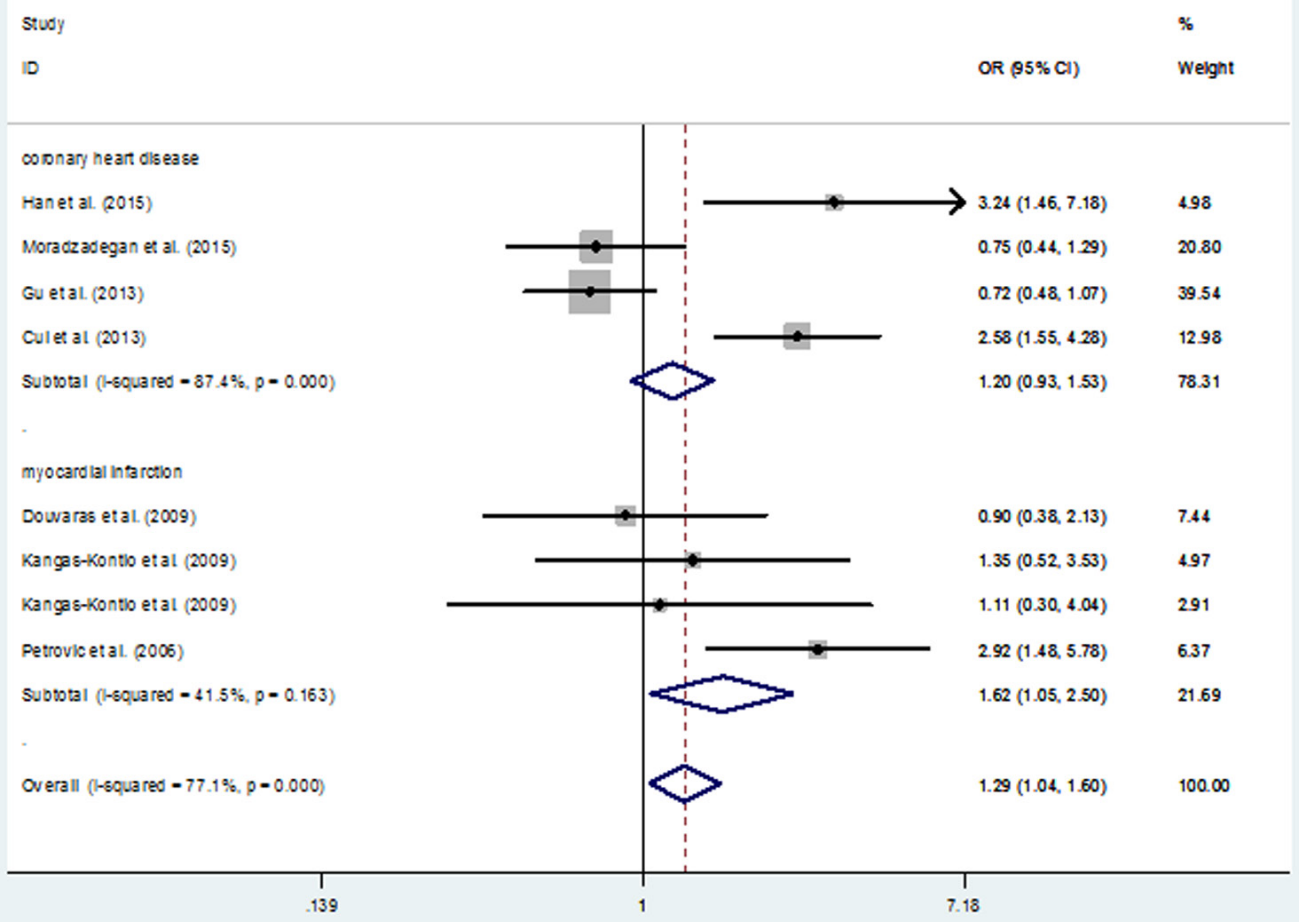
Meta-analysis for the association between VEGFA 2010963 G > C polymorphism and CHD risk (CC *vs*. GG genetic model; fixed-effects model)

For *VEGFA* rs2010963 G > C polymorphism, this SNP was not associated with risk of overall CHD (Table 7). However, in a subgroup analysis by the type of CHD, the polymorphism was associated with MI risk (CC *vs*. GG: OR = 1.62; 95% CI, 1.05 - 2.50; *P* = 0.029; CC *vs*. CG+GG: OR = 1.51; 95% CI, 1.01 - 2.27; *P* = 0.047; Table 7 and Figure 4).

**Table 7:** Meta-analysis of the *VEGFA* 2010963 G>C polymorphism and CHD

	No. of study	Allelic comparison			Homozygote comparison			Dominant comparison			Recessive comparison		
		OR(95%CI)	*P*	P(Q-test)	OR(95%CI)	*P*	P(Q-test)	OR(95%CI)	*P*	P(Q-test)	OR(95%CI)	*P*	P(Q-test)
Overall	8	1.17(0.93,1.47)	0.182	<0.001	1.43(0.87,2.35)	0.160	<0.001	1.12(0.87,1.45)	0.379	0.006	1.41(0.93,2.14)	0.102	0.001
Ethnicity													
Asians	3	1.31(0.83,2.06)	0.242	<0.001	1.76(0.65,4.78)	0.269	<0.001	1.25(0.88,1.78)	0.210	0.061	1.65(0.64,4.25)	0.297	<0.001
Caucasians	5	1.09(0.82,1.43)	0.562	0.019	1.25(0.70,2.21)	0.454	0.042	1.02(0.68,1.54)	0.922	0.008	1.25(0.92,1.69)	0.146	0.450
Type of CHD													
MI	4	1.17(0.87,1.59)	0.306	0.062	**1.62(1.05,2.50)**	**0.029**	0.163	1.15(0.74,1.78)	0.527	0.038	**1.51(1.01,2.27)**	**0.047**	0.604
Non-MI	4	1.18,0.83,1.67	0.368	<0.001	1.41(0.66,3.00)	0.376	<0.001	1.09(0.77,1.56)	0.625	0.013	1.43(0.74,2.75)	0.287	<0.001

Abbreviations: MI: myocardial infarction;

CHD: coronary heart disease

### Tests for publication bias

**Figure 5:**
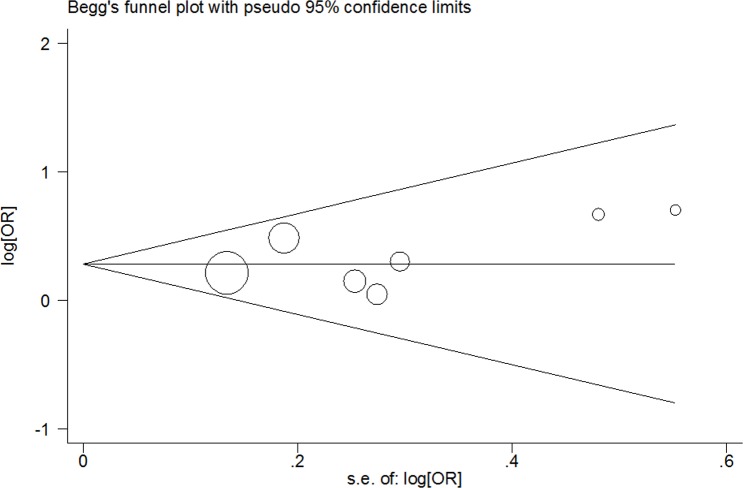
Begg's funnel plot of meta-analysis for the association between VEGFA rs699947 C > A polymorphism and CHD risk (AA+CA *vs*. CC genetic model)

**Figure 6:**
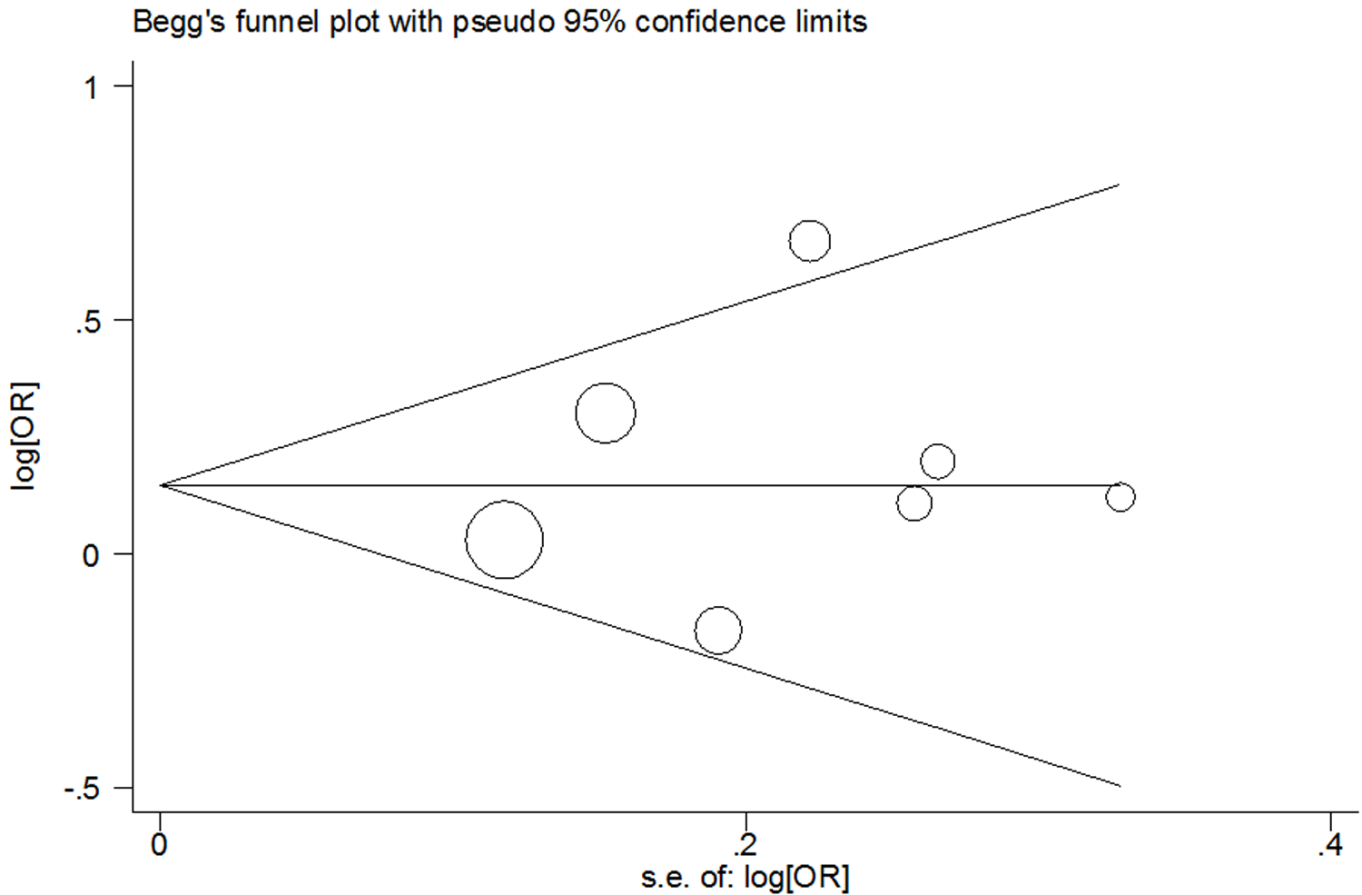
Begg's funnel plot of meta-analysis for the association between VEGFA rs3025039 C > T polymorphism and CHD risk (T *vs*. C genetic model)

**Figure 7:**
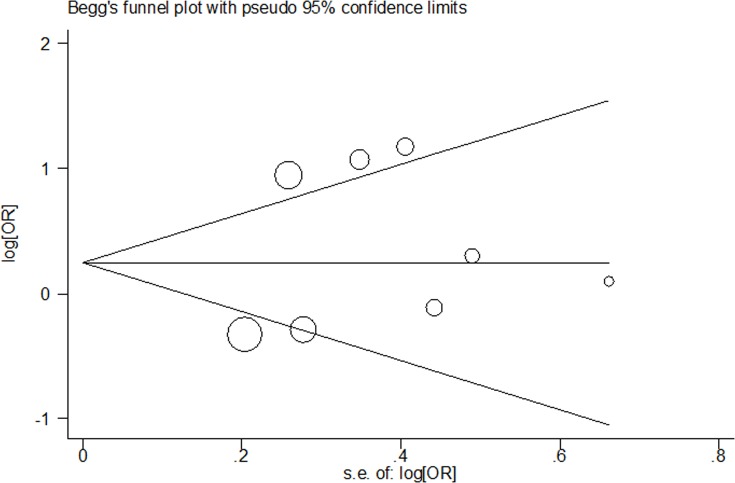
Begg's funnel plot of meta-analysis for the association between *VEGFA* rs2010963 G > C polymorphism and CHD risk (CC *vs*. GG genetic model)

The shape of Begg's funnel plot test was symmetrical for *VEGFA* rs699947 C > A, rs3025039 C > T and rs2010963 G > C polymorphisms (rs699947 C > A polymorphism: A *vs*. C: Begg's test *P* = 0.764; AA *vs*. CC: Begg's test *P* = 0.368; AA+CA *vs*. CC: Begg's test *P* = 0.548 and AA *vs*. CC+CA: Begg's test *P* = 0.230; rs3025039 C > T polymorphism: T *vs*. C: Begg's test *P* = 0.764; TT *vs*. CC: Begg's test *P* = 1.000; TT+CT *vs*. CC: Begg's test *P* = 0.548 and TT *vs*. CT+CC: Begg's test *P* = 1.000; rs2010963 G > C polymorphism: C *vs*. G: Begg's test *P* = 1.000; CC *vs*. GG: Begg's test *P* = 1.000; CC+GC *vs*. GG: Begg's test *P* = 1.000 and CC *vs*. GG+GC: Begg's test *P* = 0.902; Figure 5, Figure 6 and Figure 7). The statistical results of Egger's test still demonstrated there were no evidence of bias for these two SNPs (rs699947 C > A polymorphism: A *vs*. C: Egger's test *P* = 0.627; AA *vs*. CC: Egger's test *P* = 0.257; AA+CA *vs*. CC: Egger's test *P* = 0.394 and AA *vs*. CC+CA: Egger's test *P* = 0.356; rs3025039 C > T polymorphism: T *vs*. C: Egger's test *P* = 0.598; TT *vs*. CC: Egger's test *P* = 0.783; TT+CT *vs*. CC: Egger's test *P* = 0.475 and TT *vs*. CT+CC: Egger's test *P* = 0.660; rs2010963 G > C polymorphism: C *vs*. G: Egger's test *P* = 0.608; CC *vs*. GG: Egger's test *P* = 0.445; CC+GC *vs*. GG: Egger's test *P* = 0.899 and CC *vs*. GC+GG: Egger's test *P* = 0.318).

### Tests for sensitivity analyses

**Figure 8:**
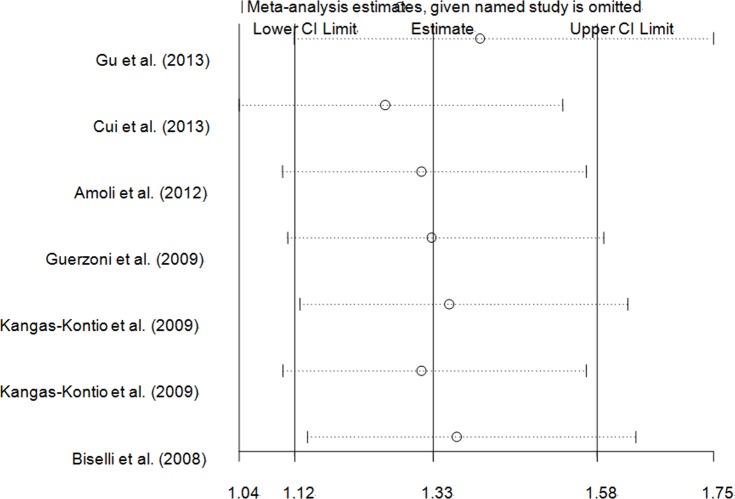
Sensitivity analysis of the overall CHD meta-analysis for *VEGFA* rs699947 C > A polymorphism

**Figure 9:**
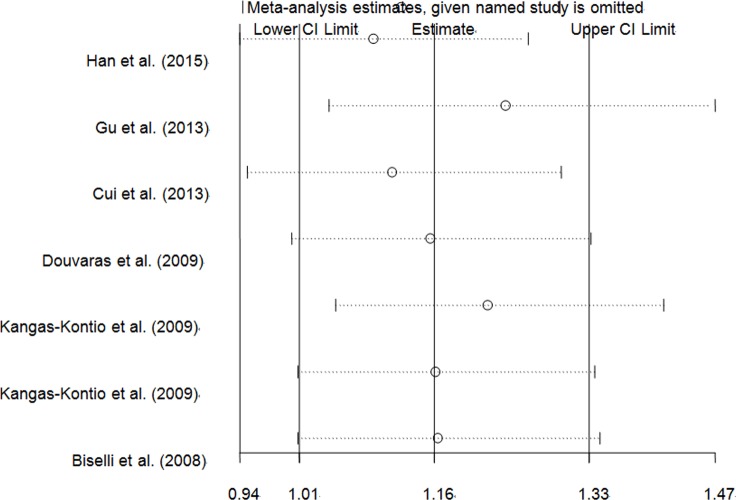
Sensitivity analysis of the overall CHD meta-analysis for *VEGFA* rs3025039 C > T polymorphism

**Figure 10:**
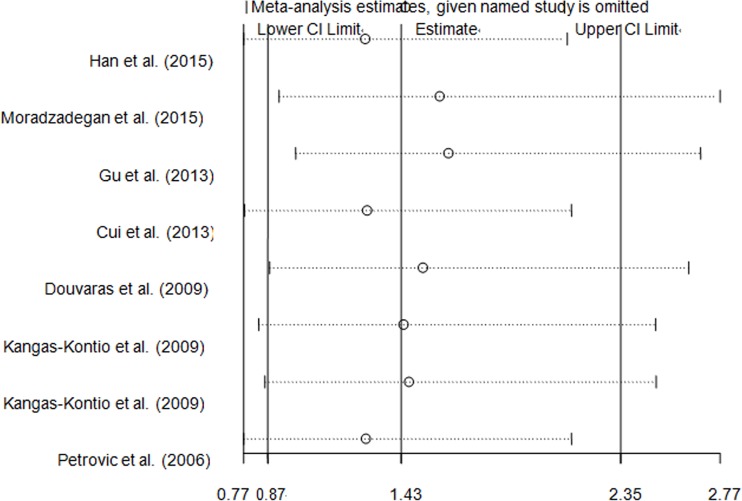
Sensitivity analysis of the overall CHD meta-analysis for *VEGFA* rs2010963 G > C polymorphism

An independent study involved in the present pooled-analysis was omitted each time to assess the influence of the data-set on the pooled ORs, and the exclusion of anyone did not materially alter the corresponding pooled ORs (Figure 8, Figure 9 and Figure 10, data not shown).

### Tests for heterogeneity

In some genetic models, we found significant heterogeneity across studies in the present meta-analysis for *VEGFA* rs699947 C > A, rs3025039 C > T and rs2010963 G > C polymorphisms. Type of CHD and ethnicity were defined as characteristics for evaluation of potential heterogeneity. Results of subgroup analyses demonstrated that studies conducted in Asians and non-MI subgroups may contribute to the major source of heterogeneity for *VEGFA* rs699947 C > A, rs3025039 C > T and rs2010963 G > C polymorphisms.

### Results of quality assessment

We used Newcastle-Ottawa Quality Assessment Scale to assess the quality score of the eligible studies. When scores ≥ 7 stars, the study was considered as high-quality. The results indicated that all included studies were high-quality, suggesting the reliability of our findings (Table 8).

**Table 8:** Quality assessment of the included studies

Study	Year	Selection				Comparability of the cases and controls	Exposure			Total stars
		Adequate case definition	Representativeness of the cases	Selection of the controls	Definition of Controls		Ascertainment of exposure	Same ascertainment method for cases and controls	Non-Response rate	
Han *et al*.	2015	*	*	—	*	**	**	*	—	8
Moradzadegan *et al*.	2015	*	*	—	*	**	*	*	—	7
Gu *et al*.	2013	*	*	—	*	**	**	*	—	8
Cui *et al*.	2013	*	*	—	*	**	*	*	—	7
Amoli *et al*.	2012	*	*	—	*	**	**	*	—	8
Guerzoni *et al*.	2009	*	*	—	*	**	**	*	—	8
Douvaras *et al*.	2009	*	*	—	*	**	**	—	—	7
Kangas-Kontio *et al*.	2009	*	*	*	*	**	**	*	—	9
Kangas-Kontio *et al*.	2009	*	*	*	*	**	**	*	—	9
Biselli *et al*.	2008	*	—	—	*	**	**	*	—	7
Petrovic et al.	2006	*	*	—	*	**	**	*	—	8

## DISCUSSION

Besides environmental risk factors (e.g. smoking, drinking, and sedentary lifestyle *et al*.), multiple evidences support a vital role of genetics in determining susceptibility for CHD. The involvement of *VEGFA* in inflammation and neovascularization may underlie the major mechanism responsible for the association between *VEGFA* genotypes and risk of CHD. Recently, several investigations on the molecular epidemiology considering on the correlation of *VEGFA* polymorphism with CHD risk were performed; however, the findings remained conflicting. With respect to *VEGFA* polymorphisms, a recent systemic review and meta-analysis with small sample sizes on this issue did not suggest any association between *VEGFA* rs699947 C > A polymorphism and risk of CHD [14]. After that, some case-control studies reported that rs699947 C > A polymorphism in *VEGFA* gene have been implicated in CHD risk, especially in Asians. Thus, we conducted a meta-analysis involving a total of 2097 CHD cases and 2867 controls subjects from ten publications including 22 trails to assess the potential associations between two commonly functional SNPs (rs699947 C > A, rs3025039 C > T and rs2010963 G > C) in *VEGFA* gene and CHD risk.

For *VEGFA* rs699947 C > A polymorphism, seven independent studies focusing on the relationship of this SNP with CHD risk were included. A recent case-control study has reported positive signals of *VEGFA* rs699947 C > A polymorphism with risk of CHD [21]; contrastingly, others showed the variants of *VEGFA* rs699947 C > A polymorphism did not influence risk of CHD [19, 20, 22–24]. As shown in Table 4, *VEGFA* rs699947 C > A polymorphism was identified to be associated with the development of CHD. The A allele carriers indicated higher CHD susceptibility in comparison with the C allele carriers. In subgroup analyses by ethnicity, the similar association was found among Asians, but not Caucasians. Our results were consistent with the findings of a previous meta-analysis [14]. A previous study indicated the expression levels of VEGF mRNA in CHD patients carrying the VEGF rs699947 AA genotype were significantly lower than those who carried the VEGF rs699947 AC or CC genotypes [29]. This study also suggested that CHD patients carrying the VEGF rs699947 A allele might have more chances in developing better coronary collaterals [29]. Gokkusu *et al*. reported that VEGF might be a cardio-protective factor [30]. In this study, we found that *VEGFA* rs699947 C > A polymorphism was correlated with increased risk of CHD, suggesting the presence of the A allele, which was associated with lower expression of VEGF mRNA and activity, might lead to the increased risk of CHD.

Rs3025039 C > T polymorphism locates on the 3′-UTR region of *VEGFA* gene. Thus, it may regulate post-transcription and then influence gene expression. *VEGFA* rs3025039 C > T polymorphism was well known to influence the secreted levels of VEGFA protein and has been identified to have overt association in most studies [31]. This SNP exhibited a very strong association with epithelial ovarian cancer status and poorer prognosis [31]. A prior study indicated this 3′-UTR polymorphism was associated with the occurrence and severity of diabetic nephropathy [32]. Recently, several case-control studies focused on the association between *VEGFA* rs3025039 C > T polymorphism and CHD risk. Han *et al.* reported that *VEGFA* rs3025039 CT genotype and C allele appeared to be a genetic risk factor for CHD [26]. Cui *et al*. also found *VEGFA* rs3025039 C > T polymorphism conferred a borderline increased risk to CHD [21]. As demonstrated in Table 5, the combined evidence suggested that *VEGFA* rs3025039 C > T polymorphism was a risk factor for overall CHD. In a subgroup analysis by ethnicity and the type of CHD, a borderline increased risk to CHD was also found in Asians and non-MI subgroups (*P* = 0.089 and *P* = 0.065, respectively). These findings demonstrated the presence of the T allele may alter mRNA and secreted levels of VEGFA protein and then led to the increased risk of CHD.

Rs2010963 G > C polymorphism is located in the 5′-untranslated region in *VEGFA* gene. According to previous reports, rs2010963 G > C polymorphism was a genetic marker of microvascular complications in cases with type 2 diabetes [33–35]. Compared to those with *VEGFA* GG and GC genotypes, a remarkably higher VEGF serum level was found in healthy individuals with the *VEGFA* rs2010963 CC genotype [33, 36]. The CC genotype of the rs2010963 G > C polymorphism has been demonstrated to be related to heart failure induced by acute myocardial infarction [25]. Several studies have investigated the association between *VEGFA* rs2010963 G > C polymorphism and CHD risk. After meta-analyses in our study, we concluded that the CC genotype of the polymorphism may increase risk of MI.

Similar to other meta-analyses, some potential limitations of our meta-analysis should be acknowledged. First, although bias tests showed there was no significant publication bias in our meta-analysis and a comprehensive literature search was well designed, it is likely that certain unpublished studies might be overlooked. Second, the association of *VEGFA* rs699947 C > A, rs3025039 C > T and rs2010963 G > C polymorphisms with risk of CHD was assessed based on unadjusted estimates. If the detailed data of individuals were available, a more precise meta-analysis could be carried out. Third, for lack of individual-level data, we did not conduct a further analysis to assess any potential interactions between gene-gene and gene-metabolic traits. Finally, significant heterogeneity between the eligible studies for *VEGFA* rs699947 C > A, rs3025039 C > T and rs2010963 G > C polymorphisms was found. Our results should be interpreted with very cautions.

In conclusion, our findings indicate that *VEGFA* rs699947 C > A, rs3025039 C > T and rs2010963 G > C polymorphisms may be risk factors for the development of CHD. As the participants in some subgroup are currently limited, further well-designed studies with larger sample size to investigate the role of these loci are needed. Moreover, interactions of gene-gene and gene-environment should not be ignored.

## MATERIALS AND METHODS

### Search strategy

Genetic association publications published before the end of November 15, 2016 on CHD and polymorphisms in *VEGFA* gene were retrieved through a search of PubMed and EMBASE online databases with keywords: (vascular endothelial growth factor-A or VEGFA) and and (polymorphism or variant or SNP) and (coronary artery disease or CAD or coronary heart disease or CHD or myocardial infarction or MI). All bibliographies cited in eligible publications, reviews and meta-analysis were examined to retrieve the potential publications.

### Inclusion and exclusion criteria

The major criteria of eligible studies were: (a) studies focused on the relationship of *VEGFA* rs699947 C > A, rs3025039 C > T and rs2010963 G > C polymorphisms with CHD risk; (b) sufficient data were presented to determine the odds ratios (ORs) with their 95% confidence intervals (CIs) and *P* value, and (c) the genotyping method, equipment, and protocols used or provided reference were described in publication. Accordingly, publications providing insufficient data, CHD treatment, not case-control design, overlapping data, reviews and meta-analysis were excluded.

### Data extraction

Two authors (Y. Wang and Q. Huang) reviewed and collected information independently from eligible studies in accordance with the major criteria for inclusion and exclusion mentioned above. The following data: the surname of first author, year of publication, country, ethnicity of the participants, type of CHD [myocardial infarction (MI) or non-MI], genotyping method as well as allele and genotype frequencies, were entered into a database. In case of conflicting evaluations, disagreements over study/data inclusion were resolved by a discussion among all reviewers.

### Quality assessment

The Newcastle-Ottawa Quality Assessment Scale was harnessed to assess the quality score of the eligible studies. And scores ≥ 7 stars were considered as high-quality study [15].

### Statistical analysis

A Goodness-of-fit chi-square calculator (http://ihg.gsf.de/cgi-bin/hw/hwa1.pl) was used to examine the deviation from HWE in controls. The strength of correlation between SNPs in *VEGFA* gene and CHD risk was assessed by ORs with the corresponding 95% CIs. Type of CHD (MI or non-MI) and ethnicity were considered as characteristics for evaluation of potential heterogeneity. Ethnicity group was defined as Asians and Caucasians. We used Chi-square based *I*^2^-statistic test and Q statistical test to analyze the potential heterogeneity among the studies. *P* < 0.10 or *I*^2^ > 50% indicates high heterogeneity, random-effects model (the DerSimonian and Laird method) was used to calculate the pooled ORs and CIs [16]; otherwise, the fixed-effects model (the Mantel-Haenszel method) was used [17]. Funnel plots and Egger's regression test were harnessed to diagnose the potential publication bias [18], and a *P* < 0.1 was defined as statistical significance. Sensitivity analysis, which assessed the influence of each independent study on the pooled ORs with their corresponding 95% CIs, was also carried out to evaluate the stability of our results. All *P* values were defined as two-sided at the *P* = 0.05 level. All data analysis was performed with Stata 12.0 software for windows (Stata Corporation, College Station, TX).
